# The experience of suicidal patients with inpatient nursing care

**DOI:** 10.1192/j.eurpsy.2023.162

**Published:** 2023-07-19

**Authors:** T. Maes

**Affiliations:** ^1^Psychiatric Care, GZA Hospitals; ^2^Faculty of medicine and health services, University Antwerp, Antwerp, Belgium

## Abstract

**Abstract:**

Worldwide, suicidal patients are treated in residential care. The used interventions and support systems during inpatient care are important in suicide prevention. Nurses are asking for guidelines on how to provide care for suicidal depressed patients. The aim of this study is to explore useful processes during the nursing care for suicidal patients. These processes are identified by exploring the suicidal patient’s experiences with nursing care. We have developed a category system of helpful processes and interventions that are observed in nursing care for suicidal patients. The category system contains four core themes; nurse behavioral interventions, nurse attitudes, and nurse conversational intervention and environment.

**Disclosure of Interest:**

None Declared

